# “Keeping family matters behind closed doors”: healthcare providers’ perceptions and experiences of identifying and managing domestic violence during and after pregnancy

**DOI:** 10.1186/s12884-017-1520-4

**Published:** 2017-09-22

**Authors:** Mary McCauley, Jennifer Head, Jaki Lambert, Shamsa Zafar, Nynke van den Broek

**Affiliations:** 1 Centre for Maternal and Newborn Health, Liverpool School of Tropical Medicine, Liverpool, UK; 2 Child Advocacy International, Islamabad, Pakistan

**Keywords:** Domestic violence, Healthcare providers, Antenatal care, Postnatal care, Pregnancy, Maternal morbidity

## Abstract

**Background:**

Violence against women is an international public health concern and a violation of women’s rights. Domestic violence can first occur, and increase in frequency and severity, during and after pregnancy. Healthcare providers have the potential to identify and support women who experience domestic violence. We sought to investigate the knowledge and perceptions of domestic violence among doctors who provide routine antenatal and postnatal care at healthcare facilities in Pakistan. In addition, we explored possible management options from policy makers, and enabling factors of and barriers to the routine screening of domestic violence.

**Methods:**

Semi-structured key informant interviews were conducted with doctors (*n* = 25) working in public and private hospitals and with officials involved in domestic violence policy development (*n* = 5) in Islamabad, Pakistan. Transcribed interviews were coded and codes grouped into categories. Thematic framework analysis was undertaken to identify emerging themes.

**Results:**

Most doctors have a good awareness of domestic violence and a desire to help women who report domestic violence during and after pregnancy. Enabling factors included doctors’ ability to build rapport and trust with women and their suggestion that further education of both healthcare providers and women would be beneficial. However, domestic violence is often perceived as a “family issue” that is not routinely discussed by healthcare providers. Lack of resources, lack of consultation time and lack of effective referral pathways or support were identified as the main barriers to the provision of quality care.

**Conclusions:**

Doctors and policy advisors are aware of the problem and open to screening for domestic violence during and after pregnancy. It is suggested that the provision of a speciality trained family liaison officer or healthcare provider would be beneficial. Clear referral pathways need to be established to provide quality care for these vulnerable women in Pakistan.

## Background

Violence against women is an international public health concern and is a violation of women’s rights [1]. The Sustainable Development Goal number 5 (SDG 5) is to achieve gender equality and empower all women and girls, with the United Nation (UN) specific targets: (5.1) to end all forms of discrimination and (5.2) to eliminate all forms of violence against all women and girls by 2030 [2]. Domestic violence occurs across all countries, cultures, religions, socioeconomic status, and ages, with an estimated one in three women suffering domestic violence during their lifetime worldwide [1].

The UN defines violence against women as "any act of gender-based violence that results in or is likely to result in physical, sexual or mental harm or suffering to women, including threats of such acts, coercion or arbitrary deprivation of liberty, whether occurring in public or in private life" [3]. It is recognised that domestic violence can often first occur, and increase in frequency and severity for women during and after pregnancy [4].

Domestic violence during pregnancy is associated with poor health outcomes for both the woman and her unborn baby including maternal injury or death; and pregnancy complications such as placental abruption, premature rupture of membranes, preterm labour, low birth weight and stillbirth [5–11]. Domestic violence also has life-long negative implications for a woman’s general health including physical (chronic pain, migraines) and psychological (anxiety, depression, post-traumatic stress disorder) consequences [6, 8, 10, 11]. Repeated healthcare attendance for non-related reasons commonly occurs where a woman is experiencing domestic violence [11]. Domestic violence is still considered a taboo subject in many countries, including Pakistan, resulting in a hidden burden of ill-health in women [12].

A current international priority is that all women have the right to the highest attainable standard of health and well-being [13]. It is imperative that healthcare providers provide good quality care for women that goes beyond simply the physical aspects of health and is inclusive of psychological and social well-being [13].

The high prevalence of domestic violence and its detrimental impact on the health and well-being of mothers during and after pregnancy has resulted in health policy development in high income countries (HIC) such as the United Kingdom (UK) where screening for domestic violence is routinely conducted during antenatal care by a trained healthcare provider [7]. The United States of America (USA) and Canada also endorse routine screening of domestic violence and report improved health outcomes for pregnant women and a decrease in domestic violence throughout the pregnancy [8, 14].

Globally, 83% of women access antenatal care (ANC) at least once during pregnancy and this currently represents a missed opportunity for HCP working in low- and middle-income countries (LMIC), who are in a unique position with the potential to identify abuse during pregnancy [15–17]. Currently, there is a lack of implementation of guidelines for the detection and management of women who experience domestic violence during and after pregnancy in LMIC. This study sought to investigate the knowledge and perceptions of domestic violence among doctors who provide routine antenatal and postnatal care at healthcare facilities in Pakistan. In addition, we explored possible management options, enabling factors of and barriers to routine screening of domestic violence; and how to translate these recommendations into practice from the perspective of policy makers.

## Methods

### Study design and setting

A qualitative descriptive design was used. Data collection was conducted using semi-structured key informant interviews (KII) with doctors working in the obstetric departments of public and private hospitals in the Islamabad Capital Territory in Pakistan in May 2016. A sub-sample of policy advisors and researchers were included. All KII were held in a private location of the participants’ choice at their relevant workplace, in the hospitals or offices.

### Participants

Doctors were included if they provided routine antenatal and postnatal care in secondary level healthcare facilities. Policy makers, policy advisors and researchers were included if they were actively involved in advocacy and the development of domestic violence guidelines. This enabled triangulation of the data and broadened the scope of the topic. Participants were chosen purposively, based on their involvement with an ongoing maternal health research project assessing maternal morbidity with Child Advocacy International (CAI) in partnership with the Liverpool School of Tropical Medicine (LSTM). Snowballing and opportunistic techniques were employed to identify the participants involved in policy and research. Participants were recruited sequentially until saturation was met.

### Topic guide

A topic guide was developed to guide the KII and was piloted in the United Kingdom (UK) with several doctors and clinical researchers from LMIC. The topic guide was subsequently refined to improve its quality; for example, the introduction was amended to ensure that the participants were aware that we sought to assess their general views and not their own personal experiences (if any) of domestic violence. The topic guide served as a flexible tool to facilitate the interviewers in obtaining the participants’ answers whilst ensuring that the interview remained on topic. The topic guide also acted as a cue to ask more probing questions to further understand participants’ beliefs and perceptions. In addition to demographics, the topic guide included five main subjects: 1) the level of rapport and trust between healthcare providers and women attending for routine antenatal or postnatal care, 2) awareness of domestic violence during and after pregnancy 3) views on routine screening for domestic violence 4) current management and referral options for women who report domestic violence and 5) suggestions for change to be able to provide a better quality of care.

### Data collection

Prior to interview, all eligible participants were approached and given verbal and written information regarding the study including a brief overview of the research aims and interview questions. An interview appointment was then scheduled at a time convenient for the participant. All participants were interviewed in English, with the average interview lasting 30 min. Interviews were conducted face-to-face, recorded on a digital recording device and transcribed upon completion. Credibility was established with the use of triangulation data from the policy makers’ interviews. All efforts were made to emphasise confidentiality and anonymity to ensure the participants were confident in giving honest answers. Interviewing participants with varied levels of experience, who have spent time in different clinical settings and from numerous areas within Pakistan, enabled generalisability to an extent.

### Analysis

Transcribed interviews were initially open coded and then reviewed by a second researcher for sense checking and to avoid bias. Codes were identified and grouped into categories by the first researcher and then reviewed by a second reviewer, enabling the first abstraction of data [18]. Thematic framework analysis of the categories was then undertaken by the first researcher and independently by a second researcher. The separate results were then brought together and refined to agree on the key themes. This strengthened the results and helped to remove potential bias [18].

### Ethics

Full ethical approval was granted by the Liverpool School of Tropical Medicine, UK (LSTM14.025) in addition to the National Bioethics Committee in Pakistan (4-87/14/NBC-159/RDC/1850). Written informed consent was obtained from all participants of the study.

## Results

### Participants’ characteristics

Key informant interviews were conducted with 26 doctors and five policy advisors and researchers. Only one person refused to take part. One of the interviewee’s data was not included in the analysis as during the interview it became apparent the participant did not have experience of delivering antenatal or postnatal care. Nearly all the doctors (24) were women, reflecting the preponderance of female healthcare providers in this speciality. The participating doctors varied in their level of experience with 12 having qualified within the past 2 years, four with 3–10 years’ experience and 10 with more than 10 years’ experience. The participants worked in different settings with four based in private hospitals, 16 in government funded hospitals and 6 who currently worked in both settings. Four doctors had previous experience of working in a rural area. Three out of the five officials were female: three worked in policy development, one in research and one in a non-governmental organisation (Table 1).

**Table 1:** Characteristics of healthcare providers

Characteristic		Doctors
Sex	Female	24
	Male	1
TOTAL		25
Place of work	Hospital, private	4
	Hospital, public	15
	Both private and public hospitals	6
TOTAL		25
Experience	Trainee general doctor	11
	Junior Obstetricians	4
	Senior Obstetricians	10
TOTAL		25

### Emerging themes

The main emerging themes included: confidence in their ability to build rapport and trust with women; domestic abuse as a taboo subject and not a health priority; perceptions regarding who suffers domestic violence; need for further education; lack of time and workload constraints; and lack of clear and effective referral pathways and support. These are separated below into enablers (opportunities) and barriers (challenges). In addition, we outline proposed solutions.

### Enablers

Key themes that would enable an improved approach to women who suffer domestic violence include: healthcare provider confidence in their ability to develop rapport with women, knowledge about domestic violence, and some clinical experience of approaching the subject of domestic violence. Within these themes, there was an underlying willingness of the healthcare providers to take more action against domestic violence. However, it became clear that the barriers were too obstructive for them to do so.

#### Confidence in the ability to build rapport and trust with women

Most doctors felt that they can build a good rapport with the women they look after, had positive experiences of doing so and were confident that they could gain the trust of women. Most doctors reported that they were aware of the problem of domestic violence but do not routinely screen women. Few healthcare providers reported that women have ever disclosed domestic violence openly, and if they have, healthcare providers reported that they could only try to comfort the woman.

“Yes, it is something that is happening in our society but people will not mention it as they either feel ashamed or feared.” (Doctor)

In contrast, policy advisors expressed concern about the general lack of doctors’ identification and management of domestic violence.

“We know from research that women are being faced with the violence when they are pregnant for the first time and they go to the hospitals because there is an issue that results because of the violence…but the doctors, they do not know how to recognise the signs or how to approach a patient.” (Policy advisor)

#### Doctors wish to have further education

Most doctors were keen for further training in how to engage and counsel women regarding domestic violence and suggested this should also be integrated into future medical student undergraduate studies. This was supported by the policy makers.

“A lot of the time we are just stuck with the situation, we don’t know how to cater for this situation, how to help that woman [with domestic violence].” (Doctor)

“There should be sensitization training…this happens and this is very common, particularly in our patriarchal society were women are kind of a property of the male members, so they are not going to discuss this openly first. It is only when they come in contact (because you know that is the only person which they might be allowed to contact) with a healthcare worker. So, the healthcare workers need to be firstly trained and sensitized to this issue.” (Policy advisor)

More senior doctors reported that confidence in the management of cases also increased with experience.

“Once you come across and you deal (with) a scenario then from the next time onwards you become more confident.” (Doctor)

#### Management of women who report domestic violence

Within the hospital setting, one referral pathway was to the psychiatric department as some doctors felt this was beneficial for the women’s subsequent depression and anxiety. Many doctors felt that this was the only option available to them, although they acknowledged it was not ideal.

“Well, over here at the hospital the most that I can do for the patient is refer her to a psychologist.” (Doctor)

One doctor explained that it was her practice to contact the police.

“We should actually tell the police that there is domestic violence. Otherwise it keeps going on and they can kill the woman. So, if it is that bad, we do tell the police.” (Doctor)

It was acknowledged that women were unlikely to seek support from the police or other formal settings, as most women were keen to avoid legal proceedings and/or were unable to leave their husbands. Women taking these steps would be considered as bringing “dishonour” upon their family and if it became known they were seeking support; this could put them in more danger.

“When we tell them that we can help them if they want the help of some NGO or police then they withdraw. They say, ‘we would face more violence if we went for help from these NGO’s or went to the police’.” (Doctor)

### Barriers

Key barriers to the identification and management of domestic violence include domestic abuse as a taboo subject, time and workload constraints, perceptions of who is at risk for domestic violence, and lack of clear referral pathways.

#### Domestic abuse as a taboo subject and the denial of problem

Most doctors were aware of the problem of domestic violence within their society but most felt it is still considered a taboo subject.

“It is quite a taboo over here…. they used to come in with bruises or something and they were always saying they fell down the stairs or something like that and they were not telling [us] the real reason, the actual reason for the bruises.” (Doctor)

“It’s just the way we are, you know the social setup, we don’t tend to open up our private things in front of people, even the doctors.” (Policy advisor)

A few doctors felt domestic violence was not a priority.

“Domestic violence I think it is not a prime issue. There are many other issues in our society which have to take priority, that’s why it is not. This topic is not so important.” (Doctor)

A few doctors who worked in private hospitals commented that domestic violence was not a problem in women attending for private health care.

“Domestic violence is not that common in the group of patients I see because I usually see girls from good, educated, well off families…but in lower classes, less educated, less resources, yes I would say there it is a problem.” (Doctor)

Most doctors felt that domestic violence was more of a problem in rural, poorer areas.

“Most of the problem, most of the women…come from the rural area. In the urban area, the females are particularly fine they are doing their own work, they are standing on their own feet…they are earning.” (Doctor)

#### Lack of time

Doctors working in public hospitals reported that their workload was too heavy and they did not have the time to enquire about aspects of health other than the basic medical information or resources to help women.

“There are too few doctors and too many patients, so a doctor can only give her five minutes or less than five minutes. There is no time for such stories.” (Doctor)

“It is very hectic; you have to see more than 100 patients in one day…you can give only two to three minutes per patient.” (Doctor)

“A lot of times we are just stuck in this situation, we don’t know how to cater for the situation, how to help the woman…most of the time we ignore it… loads of doctors basically ignore the problem, even if they know it is there, because they know they can’t help.” (Doctor)

#### Concerns for personal safety

Several doctors reported that they had witnessed incidents where a healthcare provider had been targeted and threatened by the woman’s family because they had helped a woman seek help in case of domestic violence.

“If you interfere in the lives of the women, the husband comes along, the husband says, ‘Stop, it is my personal issue, who are you to tell me how it is going’. The husband does not come alone; he comes with a lot of people. There have been incidents where doctors have been beaten up. When they come… it can turn out really bad.” (Doctor)

Similarly, in the event of legal proceedings, a doctor would have to testify in court and this was deemed very dangerous for doctors.

“We can’t go to the courts, it’s so unsafe, our family wouldn’t allow us to go to the courts with the whole environment over there, we need to have somebody to protect us…sitting in a hospital doesn’t make us protected at all, I mean, anybody can just come and point a gun at us.” (Doctor)

### Solutions

Most participants felt that doctors alone should not be responsible for counselling and managing women who report domestic violence during and after pregnancy. It was suggested that a different cadre of healthcare provider (such as a nurse or midwife) working in the hospital should receive specialised training. Doctors could refer women who experienced domestic violence. It was recognised that a specially trained healthcare provider would likely be more accessible, have more time and ability to follow-up women who needed this and could thus provide long term support to women who experience and report domestic violence.

“So, if there is a third person or persons created [to deal with this]. Then, when I have got a [woman with] domestic violence, I can say you should go to this counter or this room, there is this person sitting…they are going to listen and there is a special area for it, there is a number [you can call] and they are linked up. Then that can be helpful, that could work.” (Doctor)

“The thing is that we can work only in the hospital. We can look after them in the hospital but we cannot do anything at home. So, the third party can visit the homes and they can find out whether the woman is doing well, is not doing well, if there is any domestic violence at home.” (Doctor)

Doctors were adamant that the approach needs to be culturally appropriate, highlighting that methods that have been successful in other cultures would probably not be appropriate in Pakistan. It was explained in particular that ‘keeping family matters behind closed doors’ is of great importance. Therefore, any support program must be inclusive of the entire family (including in-laws) and confidentiality must be maintained for it to become a feasible option for victims.

### Education and empowerment of women

An increase in education for women in general was highlighted as important. Many female participants reflected on their own individual empowerment through formal education and felt that any long-term public health intervention to address domestic violence would be more successful if, in addition, women received free education and became more aware of their reproductive rights.

“When you bring education to women you give power to women. In this country…there is no education for women because education gives power and this means she can go earn [income] by herself. But it [education] does not happen and when she is a weaker part of the house [hold] she must face all of this. And when it is a natural thing because when somebody knows that he is powerful the other person is not going to say anything. So, they definitely become dominant.” (Researcher)

It was suggested that men also need to be educated to help change attitudes.

“And then, teaching men to tell them that this is something not to be practiced and this is an abnormality. If they are going [to continue with domestic violence] then they are going to repeat the same thing. They will have seen their mothers get beaten they will have seen husbands beating their wives and so no this is something that has been running in families for years and years, for centuries.” (Doctor)

## Discussion

### Statement of principal findings

Many doctors caring for women during and after pregnancy have a good understanding of the problem of domestic violence and have a desire to provide support for women who report domestic violence. However, domestic violence is still largely a taboo subject and assumptions regarding who is at risk are masking the extent of the problem in everyday practice. Doctors do not routinely screen women and report feeling helpless in their attempts to support women who do report domestic violence because of a lack of time, lack of resources, and the complex cultural context in which domestic violence occurs. Education of doctors (pre-service and in-service) would be helpful to develop their confidence to undertake routine enquiry and refer appropriately. Within the context of the busy Pakistani hospital, developing a discrete, knowledgeable screening and support team alongside the routine antenatal and postnatal care services would be the best approach with the role of the doctor being to identify and refer to a specifically trained nurse or midwife for further counsel and support.

### Strengths of the study

To the best of our knowledge, this is the first study to assess the in-depth context of healthcare providers’ knowledge and experience of domestic violence during and after pregnancy from a low-income country. This study has highlighted key areas that can support future program and policy development that aim to establish routine screening, a clear effective referral pathway and support for women who report domestic violence during and after pregnancy. A range of doctors with varied levels of experience and who had worked in different levels and types of healthcare facilities were interviewed resulting in a wide spectrum of responses. Findings were also triangulated with policy advisors and researchers improving the reliability of findings. All healthcare providers and policy makers welcomed the discussion surrounding domestic violence and were keen to contribute to solutions in their settings.

### Limitations of the study

This study population included mainly female doctors who provide routine antenatal or postnatal care in public or private secondary level healthcare facilities and excludes other cadres of healthcare providers (e.g. nurse-midwives) who also provide care and may have alternative perspectives or different insights. Similarly, this study was carried out in one urban setting in Pakistan and the findings cannot be assumed to be the same in other settings.

There is a need to assess the views of community based healthcare providers who may have different perceptions and experience. Their opinions would be important to ensure a seamless hospital to home continuum of care.

### How does this study relate to other literature?

It is widely acknowledged that a healthcare provider is likely to be the first professional contact that women experiencing domestic violence will encounter during or after her pregnancy [19]. WHO have produced clinical and policy guidelines on how to respond to pregnant women who report domestic violence including identification, safety assessment and planning, communication and clinical skills, documentation and provision of referral pathways [19]. However, the feasibility of implementation and acceptability of this guidance in countries such as Pakistan is currently uncertain [19]. There have been many calls for research into the problem of domestic violence during and after pregnancy and to test potential interventions that can be integrated and implemented in resource poor settings [20].

Integrating domestic violence into undergraduate and postgraduate pre-service and in-service programs, in keeping with a more human rights and social justice oriented approach to maternal health in general would be beneficial. There is a need for reproductive health programs to be extended to incorporate domestic violence prevention programs that include men as well as focus on gender equity and women’s reproductive autonomy [6]. Any education and training packages must be initiated and endorsed at a governmental level in Pakistan, using standardised screening tools and with an emphasis on strict ethical practice considerations to increase the reliability and authority of findings [6].

There is debate as to the cadre of healthcare providers most suitable to undertake routine screening for domestic violence. In many high-income countries, specially trained midwives routinely assess, screen, support and provide further referral [21]. However, similar challenges to the ones identified in this study are acknowledged and this requires further research.

## Conclusion

Domestic violence is common among pregnant women attending for antenatal care [5]. Women are increasingly accessing care during pregnancy in Pakistan, and there is a window of opportunity now to adapt and amend available care packages to include comprehensive screening and, where needed, support for domestic violence. Currently, healthcare providers in Pakistan do not routinely screen for domestic violence. However, many healthcare providers are open to screening women for domestic violence during antenatal and postnatal care using a culturally sensitive approach and to then refer women to a specifically trained healthcare provider or family liaison officer for further counselling and support. This study provides an understanding of the complexity of factors associated with domestic violence, provides recommendations for pathways to develop programs and is useful for policy advisors in developing efficient strategies to improve the screening, detection and management of domestic violence in women during and after pregnancy in Pakistan.

## Abbreviations

ANC: Antenatal care
CAI: Child Advocacy International
HIC: High income country
LMIC: Low and middle income country
LSTM: Liverpool School of Tropical Medicine
UK: United Kingdom
UN: United Nations
WHO: World Health Organisation

## References

[1] World Health Organization (2016). Violence Against Women Factsheet.

[2] United Nations (2015). Transforming our world: the 2030 Agenda for Sustainable Development.

[3] United Nations General Assembly. Declaration on the Elimination of Violence Against Women. Proceedings of the 85th Plenary Meeting, Geneva, Dec. 20, 1993. United Nations: Geneva.

[4] Johnson JK, Haider F, Ellis K, Hay DM, Lindow SW (2003). The prevalence of domestic violence in pregnant women. BJOG.

[5] Shrestha M, Shrestha S, Shrestha B (2016). Domestic violence among antenatal attendees in a Kathmandu hospital and its associated factors: a cross-sectional study. BMC Pregn Childb.

[6] Zakar R, Nasrullah M, Zakar MZ, Ali H (2016). (2016), The association of intimate partner violence with unintended pregnancy and pregnancy loss in Pakistan. Int J Gynaecol Obstet.

[7] Fikree F, Jafarey S, Korejo R, Khan A, Durocher J (2004). (2004), Pakistani obstetricians' recognition of and attitude towards domestic violence screening. Int J Gynaecol Obstet.

[8] Nelson HD, Bougatsas C, Blazina I (2012). Screening Women for Intimate Partner Violence: A systematic review to update U.S Preventative Risk Force Recommendations. Ann Intern Med.

[9] Usta J, Antoun J, Ambuel B, Khawaja M. Involving the Healthcare System in Domestic Violence: What Women Want. Ann Fam Med. 2012;10(3):213–20. doi:10.137o/afm.1336 10.1370/afm.1336 PMC3354970 22585885

[10] Ellsberg M, Jansen HA, Heise L, Watts CH, Garcia-Moreno C (2008). Intimate Partner Violence and Women’s Physical and Mental Health in WHO Multi-Country Study on Women’s Health and Domestic Violence: An Observational Study. Lancet.

[11] Ambuel B, Hamberger LK, Guse CE, Melzer-Lange M, Phelan MB, Kistner A (2013). Healthcare can change from within. J Fam Violence.

[12] Karmaliani R, Irfran F, Bann C, McClure E, Moss N, Pasha O, Goldenberg R (2008). Domestic violence prior to and during pregnancy among Pakistani women. Acta Obstet Gynecol Scand.

[13] United Nations (2015). Every Woman, Every Child: Global Strategy.

[14] Bair-Merritt MH, Lewis-O’Connor A, Goel S, Amato P, Ismailji T, Jelley M, Lenahan P, Cronholm P (2014). Primary Care-Based Intervention for Intimate Partner Violence: A Systematic Review. Am J Prev Med.

[15] World Health Organization (2016). Antenatal Care.

[16] World Health Organization (2015). World Health Statistics 2015.

[17] Macy R, Martin S, Kupper L, Casanueva C, Guo S (2007). Partner violence among women before, during, and after pregnancy: multiple opportunities for intervention. Womens Health Issues.

[18] Ritchie J, Lewis J (2003). Qualitative research practice: a guide for social science students and researchers.

[19] World Health Organization (2013). Global and regional estimates of violence against women: prevalence and health effects of intimate partner violence and non-partner sexual violence.

[20] Devries KM, Kishor S, Johnson H, Stöckl H, Bacchus LJ, Garcia-Moreno C, Watts C (2010). Intimate partner violence during pregnancy: analysis of prevalence data from 19 countries. Reprod Health Matters.

[21] Eustace J, Baird K, Saito A, Creedy D (2016). Midwives’ experiences of routine enquiry for intimate partner violence in pregnancy. Women Birth.

